# A Population-Based Study of SARS-CoV-2 IgG Antibody Responses to Vaccination in Manitoba

**DOI:** 10.3390/vaccines12101095

**Published:** 2024-09-26

**Authors:** Brielle Martens, Paul Van Caeseele, Jared Bullard, Carla Loeppky, Yichun Wei, Joss Reimer, Lyle R. McKinnon, Souradet Y. Shaw, Jason Kindrachuk, Derek R. Stein

**Affiliations:** 1 Department of Medical Microbiology & Infectious Diseases, University of Manitoba, Winnipeg, MB R3E 0J9, Canada; 2 Department of Pediatrics and Child Health, Rady Faculty of Health Sciences, University of Manitoba, Winnipeg, MB R3A 1S1, Canada; paul.vancaeseele@gov.mb.ca; 3 Cadham Provincial Laboratory, Winnipeg, MB R3E 3J7, Canada; 4 Epidemiology & Surveillance, Manitoba Health, Seniors and Active Living, Winnipeg, MB R3B 3M9, Canada; 5 Winnipeg Regional Health Authority, Winnipeg, MB R3B 1E2, Canada; 6 Centre for the AIDS Programme of Research in South Africa (CAPRISA), Durban 4001, South Africa; 7 Department of Medical Microbiology and Immunology, University of Nairobi, Nairobi P.O. Box 19676 00202, Kenya; 8 Department of Community Health Sciences, University of Manitoba, Winnipeg, MB R3E 0W2, Canada; 9 Department of Internal Medicine, University of Manitoba, Winnipeg, MB R3E 0W2, Canada; 10 Manitoba Centre for Proteomics and Systems Biology, Health Science Centre, Winnipeg, Manitoba R3E 3P4, Canada

**Keywords:** SARS-CoV-2, COVID-19, vaccines, antibodies, IgG, serology, immunity

## Abstract

Understanding variables that influence antibody responses to COVID-19 vaccination within a population can provide valuable information on future vaccination strategies. In this population-based study, we examined the antibody responses to COVID-19 vaccination in Manitoba using residual serum specimens collected between January 2021 and March 2022 (n = 20,365). Samples were tested for spike and nucleocapsid IgG against SARS-CoV-2 using clinically validated assays. We assessed the impacts of multiple factors on post-vaccination antibody titres including type of vaccine, age, sex, geographic location, number of doses received, and timing of vaccination. Our investigation demonstrated that vaccination with one dose of Moderna mRNA-1273 elicited higher anti-spike IgG titres overall compared to Pfizer BNT162b2 vaccination, while one dose of Pfizer BNT162b2 followed by a second dose of Moderna mRNA-1273 exhibited higher titres than two doses of Pfizer BNT162b2 or Moderna mRNA-1273, irrespective of age. Age and time post-vaccination had considerable effects on antibody responses, with older age groups exhibiting lower anti-spike IgG titres than younger ages, and titres of those vaccinated with Pfizer BNT162b2 waning faster than those vaccinated with Moderna mRNA-1273 or a combination of Pfizer BNT162b2 and Moderna mRNA-1273. Antibody titres did not appear to be affected by sex or geographic location. Our results identify how factors such as age and type of vaccine can influence antibody responses to vaccination, and how antibody titres wane over time. This information highlights the importance of tailoring vaccine regimens to specific populations, especially those at increased risk of severe COVID-19 and can be used to inform future vaccination strategies, scheduling of booster doses, and public health measures.

## 1. Introduction

Severe acute respiratory syndrome coronavirus 2 (SARS-CoV-2), the etiologic agent of coronavirus disease 2019 (COVID-19), continues to have important public health impacts across the globe. The rapid geographic expansion of SARS-CoV-2 across the globe at the start of the pandemic was met with the unprecedented design, development, and clinical trial assessments of multiple vaccine candidates with public rollout beginning in late 2020. As of March 2024, more than 13.5 billion COVID-19 vaccines have been distributed globally, with over two-thirds of the global population having received the primary series of vaccine [1]. Rapid vaccine deployment and uptake were an integral component that reduced the burden of COVID-19 [2]. However, the emergence of the more transmissible and immune evasive SARS-CoV-2 variants of concern, including the Delta and Omicron lineages, as well as waning immunity among vaccinated individuals, has led to a significant increase in breakthrough infections [3,4,5,6,7]. Despite this, it remains clear that COVID-19 vaccination reduces COVID-19 disease severity and continues to be a key resource in the fight against SARS-CoV-2 [8,9].

The first COVID-19 vaccines approved for use in Canada were Pfizer BNT162b2, which was authorized by Health Canada on 9 December 2020 [10], followed by Moderna mRNA-1273 on 23 December 2020 [11]. Vaccinations in the province of Manitoba began on 16 December 2020, starting with healthcare workers working in direct contact with patients in critical care or long-term care facilities [12]. Vaccination eligibility expanded to the public in early 2021, starting with individuals ≥95 years old and First Nations people ≥75 years old [13,14]. The age of eligibility was incrementally decreased over the following months with increasing vaccine availability. Approximately 79% of Manitobans had received the primary series of COVID-19 vaccines as of September 2023 [15]. However, only 18.2% of Manitobans have received their primary COVID-19 vaccination series and all recommended booster doses as of March 2024 [16]. The current vaccination recommendations in Canada includes having received the recommended number of XBB.1.5 COVID-19 vaccines for one’s age group and health status, and having completed their primary series or received a booster dose within the last 6 months [17].

Here, we assessed the impacts of multiple demographic and vaccine-related factors on serum antibody levels from >20,000 residual specimens collected as part of the Manitoba COVID-19 Seroprevalence (MCS) study, a population-based cross-sectional study that aims to assess the prevalence of SARS-CoV-2 antibodies across Manitoba [18]. We used Health Canada-approved assays to measure the SARS-CoV-2 spike (S) and nucleocapsid (N) IgG responses within the specimens along with matched basic demographic information including age, sex, regional health authority, type of vaccine, number of doses received, and timing of vaccination.

Given the emergence of the Omicron lineage, and the ability of Omicron subvariants to evade early recognition by the immune system, this information could inform future booster dose rollouts or new vaccine formulations and regimens tailored to particular groups at increased risk for infection.

## 2. Methods

### 2.1. Study Population

This study used routinely collected population-based public health laboratory and surveillance data from Cadham Provincial Laboratory (CPL) as part of the MCS study. The MCS study is a population-based cross-sectional study initiated in April 2020 at CPL to measure the seroprevalence of SARS-CoV-2 antibodies in Manitoba. CPL routinely tests approximately 16,000 serum specimens per month from across the province. All residual specimens were eligible for inclusion in the study except those being investigated for SARS-CoV-2-related disorders such as multi-inflammatory syndrome in children and adults (MIS-C, MIS-A). Basic demographic information was collected from de-identified specimens including sex, age, and regional health authority. This study also had access to COVID-19 vaccination records for each specimen including date of vaccination and vaccine type. Additional details about the study population and MCS study were described previously [18]. Fully vaccinated participants were defined as having received two or more doses of vaccine a minimum of 14 days prior to collection. Partially vaccinated participants were defined as having one dose of a vaccine a minimum of 14 days prior to collection.

### 2.2. Specimens and Serological Assays

Serum specimens were collected between January 2021 and March 2022. Specimens were selected proportionally by percentage for sex (50:50), age (20:20:20:20:20), and regional health authority (60:10:10:10:10). Manitoba has 5 regional health authorities, which include the Winnipeg Regional Health Authority (WRHA), Northern Health Region (NHR), Southern Health-Santé Sud (SHSS), Prairie Mountain Health (PMH), and Interlake-Eastern Regional Health Authority (IERHA). Anti-spike IgG titres were measured using the DiaSorin LIAISON^®^ SARS-CoV-2 S1/S2 IgG assay, with a positive signal cut-off of ≥15 AU/mL [18,19]. Anti-nucleocapsid IgG titres were measured using the Abbott Architect SARS-CoV-2 IgG assay, with a positive signal/noise cut-off of ≥0.7 S/CO [18,20]. Only specimens aged 12 and above were included in the analyses, as most children under the age of 12 received a pediatric COVID-19 vaccine.

### 2.3. Data Analysis

Data analysis was carried out using R Studio (v. 2023.12.1+402). Figures were generated using the ggplot2, ggh4x, and ggbeeswarm packages [21,22,23]. Tables were made using the gtsummary package [24]. Statistical analysis was conducted using the rstatix, stats, and emmeans packages [25,26,27]. For the anti-S IgG analyses, specimens were grouped according to dose, age group, and vaccine type. Only specimens that had been vaccinated 14+ days before specimen collection were included. Those with positive anti-N IgG were excluded from analysis due to previous COVID-19 infection. The Kruskal–Wallis test was used to determine statistical significance, followed by Dunn’s Multiple Comparison Test using the Bonferroni adjustment for multiple comparisons. *p* values of ≤0.05 were considered significant. Linear regression was used to examine the effect of time post-vaccination on anti-spike IgG titres, followed by analysis of covariance (ANCOVA) and emmeans post-hoc tests to determine whether the regression slopes for each vaccine were significantly different from one another.

## 3. Results

### 3.1. Specimen Demographics

Specimens were collected by Cadham Provincial Laboratory (CPL), the sole public health laboratory in Manitoba, Canada as part of the MCS study [18]. A total of 20,365 residual serum specimens were included in the study. Of these, 10,308 (50.6%) were female, and 10,057 (49.4%) were male. Table 1 summarizes the demographics of study participants. The median age was 34 (IQR, 17–56 years). At the time of specimen collection, 10,422 (51%) individuals had received at least one dose of a COVID-19 vaccine. The maximum number of doses received by an individual was 6 (n = 129), and a total of 47,082 doses were administered. Pfizer BNT162b2 was the most common vaccine received, accounting for 58.0% (n = 27,288) of all vaccines administered. Moderna mRNA-1273 accounted for 22.6% (n = 10,661) of all vaccines administered. Most people who received fifth and sixth doses received bivalent formulations from either Moderna or Pfizer. The median number of weeks between receiving a first dose and blood collection was 38 (IQR, 20–55). The median number of weeks between first and second doses was 7 (IQR, 5–10), and the median number of weeks between receiving a second dose and blood collection was 34 (IQR, 19–50).

The majority of participants lived within the Winnipeg Regional Health Authority (WRHA) (57%), followed by Southern Health Santé Sud (SHSS) (14%), Prairie Mountain Health (PMHR) (12%), Interlake-Eastern Health (IEHR) (9.5%), and Northern Health (NHR) (8.1%). All specimens were tested for SARS-CoV-2 anti-nucleocapsid (N) IgG (n = 20,365), of which 7552 (37%) were positive, suggesting previous COVID-19 infection [18].

### 3.2. Vaccine Rollout and Timeline

**Figure 1:**
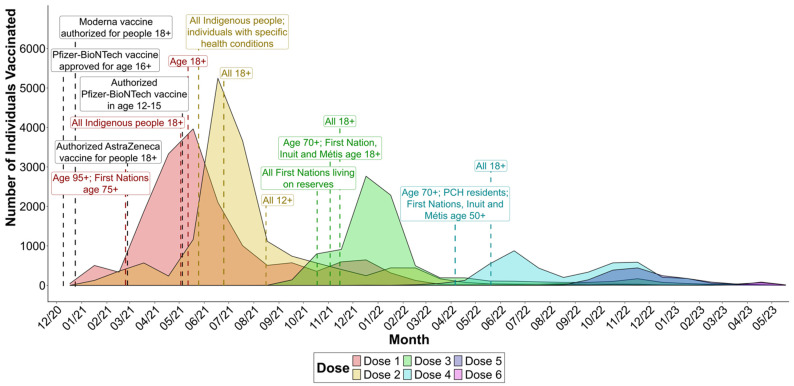
Number of COVID-19 vaccines administered by dose over time for the specimens used in this study (n = 20,365), and major changes in vaccine eligibility over time in Manitoba. Specimens were collected as part of the Manitoba COVID-19 Seroprevalence study. PCH, personal care home.

We looked at the number of COVID-19 vaccines being administered over time to see whether vaccination rates corresponded to changes in eligibility (Figure 1). The majority of first doses were administered between March and August 2021, peaking in May. The majority of second doses followed shortly after, with July 2021 being the month with the largest number of second doses received. Most third doses were received several months later, with the majority occurring between December 2021 and February 2022. The number of fourth doses received peaked in June 2022; however, this was considerably less than those who received one to three doses. Fewer individuals received fifth and sixth doses, with the number of recipients peaking in November 2022 and April 2023, respectively. These trends correspond to the changes in eligibility to receive each dose of a COVID-19 vaccine that were occurring at the time (see Table S1 for a summary of vaccine eligibility changes in Manitoba).

### 3.3. IgG Titres Vary by Vaccine Type

A total of 14,089 specimens were tested for SARS-CoV-2 anti-spike (S) IgG, of which 9322 (66%) were positive, indicating either previous COVID-19 infection or vaccination. Of those, we used the specimens that were negative for anti-N IgG to analyze anti-S IgG responses to vaccination (n = 4979). We examined whether factors such as age, sex, vaccine type, time post-vaccination, or geographic location had any impact on IgG titres. Sex and geographic location did not appear to impact titres (Figures S1 and S2), while age, vaccine type, and time post-vaccination all had significant impacts on anti-S IgG responses.

**Figure 2:**
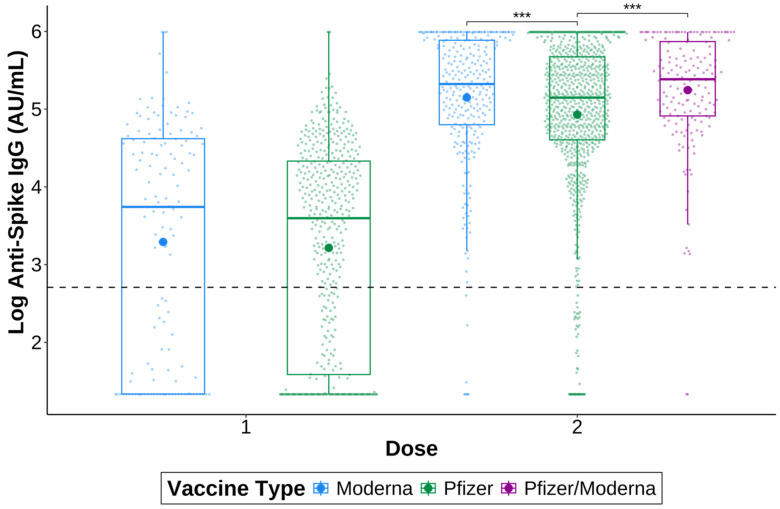
SARS-CoV-2 anti-spike IgG after the first and second doses of a COVID-19 vaccine. Specimens were vaccinated with either one dose of Pfizer BNT162b2 (green) or Moderna mRNA-1273 (blue), or with two doses of Pfizer BNT162b2 or Moderna mRNA-1273 or one dose of each (Pfizer/Moderna; purple). Only specimens negative for anti-nucleocapsid IgG were included. The larger dots are the mean. The smaller dots represent individual samples. The dashed line indicates a positive signal cut-off of 15 AU/mL. *** *p* ≤ 0.001.

To investigate whether anti-S IgG titres differed between vaccine types, specimens were grouped by whether they received a Moderna mRNA-1273 or Pfizer BNT162b2 vaccine, and the number of doses received by the time of specimen collection (Figure 2). There were no significant differences between vaccines after one dose; however, those who received two doses of Moderna mRNA-1273, (*p* = 2.91 × 10^−4^) or one dose of Pfizer BNT162b2 followed by a dose of Moderna mRNA-1273 (Pfizer/Moderna; *p* = 2.91 × 10^−4^) had significantly higher anti-spike IgG titres than those who received two doses of the Pfizer-BioNTech vaccine.

### 3.4. IgG Titres Vary by Age

**Figure 3:**
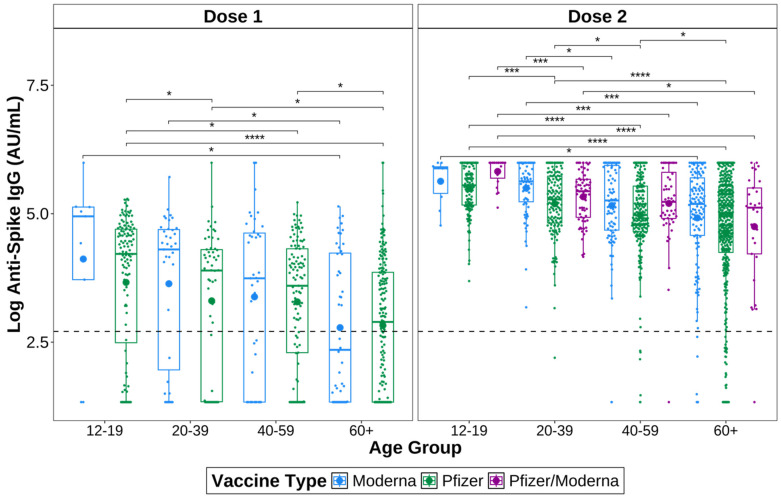
Age differences in SARS-CoV-2 anti-spike IgG after one or two doses of a COVID-19 vaccine. Only samples negative for anti-nucleocapsid IgG were included. Specimens were vaccinated with either one dose of Pfizer BNT162b2 (green) or Moderna mRNA-1273 (blue), or with two doses of Pfizer BNT162b2 or Moderna mRNA-1273, or one dose of each (Pfizer/Moderna; purple). Dunn’s test for pairwise comparisons corrected for multiple hypothesis testing using the Benjamini–Hochberg method to compare groups. The larger dots are the mean. The smaller dots represent individual samples. The dashed line indicates a positive signal cut-off of 15 AU/mL. **** *p* ≤ 0.0001, *** *p* ≤ 0.001, * *p* ≤ 0.05.

Anti-S IgG titres of different age groups were measured to investigate whether a correlation between age and anti-S IgG exists (Figure 3). IgG titres were measured 14+ days after receiving a first or second dose and before receiving a second or third dose, respectively. Only specimens negative for anti-N IgG were included, to remove those who have had a previous COVID-19 infection. Specimens were divided into the following age groups: 12–19 years, 20–39 years, 40–59 years, and 60+ years old.

In general, anti-S IgG titres tended to decline with increasing age (Figure 3). After one dose of a COVID-19 vaccine, the 12–19-year-olds had the highest anti-S IgG titres overall, regardless of the vaccine received. Of those who received Pfizer BNT162b2, the 12–19 years group had significantly higher titres than the 20–39 (*p* = 4.44 × 10^−2^), 40–59 (*p* = 1.14 × 10^−2^) and 60+ years groups (*p* = 9.79 × 10^−9^). The 20–39 (*p* = 2.02. × 10^−2^) and 40–59 years groups (*p* = 1.14 × 10^−2^) also had higher titres than the 60+ years group after one dose of Pfizer BNT162b2. Of those who received one dose of Moderna mRNA-1273, the 12–19 (*p* = 4.77 × 10^−2^) and 20–39 years groups (*p* = 4.77 × 10^−2^) had higher titres than the 60+ years group, while the 40–59 years group did not.

After two doses of a COVID-19 vaccine, 12–19-year-olds again had the highest anti-S IgG titres overall. Of those who received two doses of Pfizer BNT162b2, the 12–19 years group again had significantly higher titres than the 20–39 (*p* = 3.81 × 10^−4^), 40–59 (*p* = 1.87 × 10^−9^), and 60+ (*p* = 2.80 × 10^−17^) years groups. The 20–39 years group had higher titres than both the 40–59 (*p* = 3.07 × 10^−2^) and 60+ (*p* = 3.78 × 10^−5^) years groups, and the 40–59 years group had significantly higher titres than the 60+ years group (*p* = 4.25 × 10^−2^). This means the 60+ years group had the lowest titres overall after two doses of Pfizer BNT162b2.

After two doses of Moderna mRNA-1273, the 12–19 years group again had the highest titres of all of the age groups; however, the titres were only significantly higher than the 60+ years group (*p* = 3.40 × 10^−2^). The 20–39 years group had significantly higher titres than both the 40–59 (*p* = 3.28 × 10^−2^) and 60+ (*p* = 1.03 × 10^−4^) years groups. The 40–59 years group did not have significantly higher titres than the 60+ years group after two doses of Moderna mRNA-1273.

The anti-S IgG titres of the 12–19 years group who received one dose of Pfizer BNT162b2 followed by Moderna mRNA-1273 (Pfizer/Moderna) were also significantly higher than all other age groups (20–39, *p* = 4.21 × 10^−6^; 40–59, *p* = 2.89 × 10^−4^; 60+, *p* = 4.21 × 10^−6^). The 20–39 years group had higher titres than the 60+ years group (*p* = 4.67 × 10^−2^), but not the 40–59 years group.

As the 40–59 age group had significantly higher titres than the 60+ age group only among those who received Pfizer BNT162b2, we next compared data from those who received Moderna mRNA-1273 to those who received Pfizer BNT162b2 or heterologous Pfizer/Moderna within the same age group. There were no significant differences found between those that received Moderna mRNA-1273 or Pfizer BNT162b2 after either one or two doses within 40–59 years age group. However, slightly higher titres were found among those aged 20–39 years following one dose of Moderna mRNA-1273 compared to Pfizer BNT162b2 (*p* = 0.05). After two doses, the 12–19 years group who received heterologous Pfizer/Moderna had significantly higher titres than those who received Pfizer BNT162b2 (*p* = 3.74 × 10^−3^). The 20–39 years group who received two doses of Moderna mRNA-1273 had higher titres than those of the same age group who received Pfizer BNT162b2 (*p* = 4.36 × 10^−4^) or Pfizer/Moderna (*p* = 0.031). There were no significant differences in the 60+ age group.

### 3.5. IgG Titres Vary over Time

**Figure 4:**
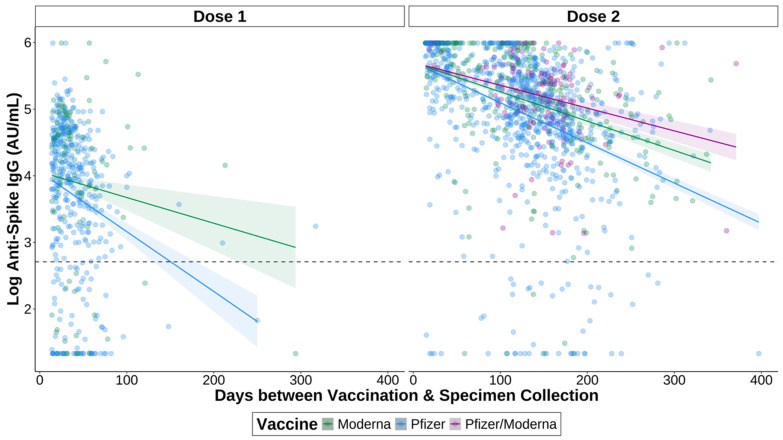
The effect of time between vaccination and specimen collection on spike IgG responses after first and second doses of a COVID-19 vaccine. Specimens were vaccinated with either one dose of Pfizer BNT162b2 (green) or Moderna mRNA-1273 (blue), or with two doses of Pfizer BNT162b2 (green) or Moderna mRNA-1273 (blue), or one dose of each (Pfizer/Moderna; purple). For the interval between Dose 1 and specimen collection, titres were measured 14+ days after receiving the first dose and before receiving a second dose. For the interval between Dose 2 and specimen collection, titres were measured 14+ days after receiving a second dose, and before receiving a third dose. Only samples negative for anti-nucleocapsid IgG were included. The shaded regions indicate standard error intervals. The dashed line indicates a positive signal cut-off of 15 AU/mL.

Finally, we assessed the impacts of time between most recent vaccination and specimen collection on anti-S IgG titres. A linear regression was used to determine whether the titres of those who waited longer between vaccination and specimen collection were lower than those whose specimens were collected sooner after vaccination (Figure 4). We found that overall titres were lower over time after one (*p* = 1.05 × 10^−5^) and two (*p* = 4.26 × 10^−41^) doses.

We also examined longitudinal IgG titres following vaccination between the two vaccine types. We found that those who received the Moderna mRNA-1273 vaccine had higher titres than those who received the Pfizer BNT162b2 vaccine over time after one dose (*p* = 0.024). After two doses, those who received a heterologous Pfizer/Moderna had the highest titres longitudinally and were significantly higher than those who received two doses of Pfizer BNT162b2 (*p* = 5.48 × 10^−6^). There was no significant difference when compared to those having received two doses of Moderna mRNA-1273. Two doses of Moderna mRNA-1273 vaccine resulted in significantly higher longitudinal IgG titres as compared to two doses of Pfizer BNT162b2 (*p* = 3.09 × 10^−4^). However, IgG titres declined over time irrespective of vaccination type.

## 4. Discussion

In this population-based study, we examined the antibody responses of Manitobans to mRNA-based COVID-19 vaccines. Our results provide a summary of vaccine-induced immunity in the Manitoba population that are likely generalizable to other national and global locations.

We compared when individuals received each dose to the changes in vaccine eligibility that were occurring in Manitoba over time. The majority of individuals were vaccinated shortly after becoming eligible for vaccination. For first doses, there was a small spike in the number of overall vaccinations received from December 2020 to February 2021, when only specific healthcare workers were eligible for the vaccine [28]. The largest spike in first doses occurred shortly thereafter as members of the general public became eligible [29]. A similar small spike in second doses which rose steadily from January until March 2021. At this time, healthcare workers and certain high-risk groups who had received a first dose were eligible for a second dose either 21 days (Pfizer BNT162b2) or 28 days (Moderna mRNA-1273) after receiving their first vaccination [30]. Following this small spike in second doses, there is a decline in second doses due to the interval between first and second doses being extended as a result of limited vaccine supply [31]. The number of second doses received peaked around June and July 2021 when the general adult population became eligible [32]. Third doses peaked around December 2021 and January 2022, shortly after the adult population became eligible [33]. However, individuals had to wait 6 months between their second and third doses, which may explain why the peak in third doses was less dramatic than first and second doses, along with fewer individuals overall receiving a third dose compared to first and second doses. Fourth doses peaked around June 2022, shortly after all adults became eligible in May [34].

One dose of the Moderna COVID-19 vaccine induced slightly higher anti-S IgG titres overall compared to the Pfizer-BioNTech vaccine, although not significantly so. Those who received two doses of Moderna mRNA-1273 or the Pfizer/Moderna combination had significantly higher titres than those who received two doses of Pfizer BNT162b2, suggesting that the Moderna mRNA-1273 vaccine resulted in only a slightly stronger antibody response than Pfizer BNT162b2. Other studies have found similar results when comparing vaccine types. Montoya et al. and Steensels et al. both found that after two doses, Moderna mRNA-1273 elicited higher anti-S IgG titres than Pfizer BNT162b2, but they did not look at heterologous vaccination [35,36]. Stuart et al. also found that heterologous vaccination with Pfizer BNT162b2 followed by Moderna mRNA-1273 generated a greater antibody response compared to two doses of Pfizer BNT162b2 [37]. Together, this demonstrates that both homologous and heterologous vaccination regimens using mRNA vaccines are safe and effective options for vaccinating against COVID-19.

We examined how sex differences affect anti-S IgG responses to COVID-19 vaccination. It has been previously established that females often develop higher antibody responses to vaccination than males, such as with influenza, measles, mumps, and rubella, among others [38]. For this reason, we wanted to know whether these sex differences occurred in mRNA vaccines for SARS-CoV-2 as well. We did not find any differences between sexes in terms of anti-S IgG. The literature on how sex impacts antibody responses to SARS-CoV-2 vaccination appears to be inconclusive. Some studies do report that females produce higher antibody titres in response to SARS-CoV-2, while others report no differences between sexes [39,40,41]. One study found no differences between sexes specifically, but did find that sex-specific comorbidities did influence antibody responses [41]. Further studies addressing sex differences in antibody responses to vaccination should ensure confounding variables such as age and comorbidities are accounted for.

We chose to look at whether differences in geographic location impacted antibody responses to COVID-19 vaccination due to the large variability in population groups between the regional health authorities in Manitoba. Different regions of Manitoba differ from one another in terms of race and ethnicity, lifestyle, education levels, and access to healthcare. The NHR in particular has a substantially higher proportion of Indigenous residents and communities than other health regions in Manitoba. The NHR also has greater difficulty accessing healthcare and other resources such as affordable food due to many communities’ remote locations [42]. The WRHA region encompasses the city of Winnipeg, the capital of Manitoba. As a result, this region has a much higher population density than the rest of the province, and its population is highly heterogeneous in regard to race and ethnicity, socioeconomic status, and lifestyle.

The SHSS region is highly diverse and is home to large Mennonite, Hutterite, Francophone, and immigrant populations. This region has the highest rate of internal migrant mobility and population growth in Manitoba [43]. Portions of SHSS have markedly lower education levels than the provincial average, such as the city of Winkler and Rural Municipality of Stanley, where over 40% of residents do not have a high school diploma [44,45]. These areas also had the lowest COVID-19 vaccination rate in Manitoba, and possibly Canada [46,47,48]. By assessing whether these geographic regions had an impact on IgG responses to COVID-19 vaccination we could infer that some of the factors unique to each region may have had an impact on IgG responses. While we did not find any differences between the regions, factors such as race and ethnicity, lifestyle, education, and access to healthcare should be examined independently for how they impact antibody responses to vaccination.

When we compared the responses to the two vaccines within the same age groups, we found that the 20–39 years age group who received Moderna mRNA-1273 or the Pfizer/Moderna combination had higher titres after two vaccine doses than those who received Pfizer BNT162b2. There were no differences between vaccines within any other age groups, and no differences after only one dose. This suggests that younger ages have greater antibody responses to Moderna mRNA-1273 than Pfizer BNT162b2, but middle-aged and older individuals respond comparably to the two vaccines. This information could help inform practitioners and patients on what type of COVID-19 vaccine may be most beneficial based on their age.

Younger age groups were found to have overall higher antibody responses to vaccination than older age groups, regardless of vaccine type. Advanced age appears to be commonly associated with lower antibody responses to both COVID-19 vaccination and infection [49,50,51,52]. This result is unsurprising given that the immune system’s ability to respond to antigens declines with age [53]. Another population-based study from British Columbia found older age to be the strongest predictor of hospital admission due to COVID-19 [54]. In Manitoba, individuals aged 60+ had the highest infection fatality ratio (IFR) in the province compared to other age groups [18]. This information highlights the importance of tailoring vaccination strategies and public health measures to protect elderly populations.

Finally, we were interested in seeing how antibody responses to vaccination wane over time. The IgG titres elicited by Pfizer BNT162b2 alone decayed the fastest compared to Moderna mRNA-1273 and Pfizer/Moderna combination. It is well documented that both antibodies and overall vaccine effectiveness decline post-COVID-19 vaccination [50,51,55,56,57,58]. This has been signified by marked decreases in antibody levels around 6 months post-vaccination with estimates for the duration of protection ranging from 5 months to 2 years [51,55,56,57,58]. Additionally, several high-risk groups, including those who are immunocompromised or of older age, have been shown to experience a greater decline in antibody levels post-vaccination [50]. It is important to note that antibody responses to SARS-CoV-2 vaccination and infection generally follow a biphasic decline. This biphasic response may explain why antibody titres declined so rapidly in the serum specimens. Several studies have found that antibody responses to SARS-CoV-2 infection decline rapidly within the first 4 months post-infection, followed by a gradual decline over several months [59,60,61,62]. Despite the initial rapid decline of antibodies post-vaccination, studies have shown that antibodies still reduce the risk of infection, and protection against severe disease remains high after 6 months, although the level of protection varies by age [51,63]. Considering that we found antibody responses to decrease rapidly over time, it would be beneficial to establish a duration of protection for high-risk groups specifically, as they may need more frequent booster doses to maintain immunity.

Overall, Moderna mRNA-1273 and the Pfizer/Moderna combination elicited higher anti-S IgG titres than Pfizer BNT162b2 after two doses. Other studies have found the Moderna mRNA-1273 to elicit significantly higher antibody responses [35,36,49,55,64] and greater effectiveness compared to Pfizer BNT162b2 [65]. One possible explanation for the stronger response produced by Moderna mRNA-1273 is a result of its higher mRNA content compared to Pfizer BNT162b2 (50 mcg vs. 30 mcg per dose for ages 12+) [66]. Additionally, heterologous vaccination against SARS-CoV-2 has been found to provide equivalent or superior antibody responses compared to homologous vaccination [37,67]. One study also found that priming with the Pfizer BNT162b2 or AstraZeneca vaccine followed by boosting wit Moderna mRNA-1273 provided superior immunogenicity than two doses of Pfizer BNT162b2, suggesting that this may reflect greater immunogenicity elicited by Moderna mRNA-1273 rather than a specific result of mixing vaccines [37]. While there may be some benefit to mixing Pfizer/Moderna, the difference in anti-S IgG titres appears to be minimal compared to two doses of Moderna mRNA-1273.

This study has several limitations. Firstly, we were unable to fully quantify the IgG titres after receiving one or more booster doses as the DiaSorin LIAISON^®^ SARS-CoV-2 S1/S2 IgG assay we used had a maximal limit of 400 AU/mL. We also did not have PCR test results for the specimens used to rule out active SARS-CoV-2 infection. Specimens were assumed to be negative for previous COVID-19 infection if they were negative for anti-N IgG. It is possible that some specimens may have had a previous COVID-19 infection and had anti-N IgG titres that were below the cut-off for a positive result. While we wanted to examine the titres of those first vaccinated with Moderna mRNA-1273 followed by Pfizer BNT162b2, we did not have enough specimens to properly compare those specimens to the other vaccine regimens. In addition, longitudinal data was not collected to examine an individual’s antibody response over time. Using longitudinal data would likely provide a more accurate representation of how antibody levels change over time within an individual. We were also unable to examine how factors such as race and ethnicity, lifestyle, education, and access to healthcare independently impact antibody responses to vaccination. Additionally, some studies have found antibody titres to be negatively impacted by body mass index (BMI) [68,69]; however, we did not have access to height and weight data for the specimens used in the study and were therefore unable to assess whether BMI had an impact on antibody responses to SARS-CoV-2 vaccination. Antibody responses are only one component of the immune response induced by vaccination. While we were unable to look at cellular immune responses to vaccination, they are an important defense mechanism, and further studies examining the effect of COVID-19 vaccination on cellular immunity at a population level would be of benefit.

## 5. Conclusions

Overall, we found that the Moderna mRNA-1273 vaccine and Pfizer/Moderna combination generated higher anti-S IgG titres than the Pfizer BNT162b2 vaccine in an analysis of more than 20,000 samples acquired within Manitoba. Additionally, increased age was associated with lower antibody titres, and antibody responses appear to decline rapidly after vaccination, highlighting the need for regular booster doses, especially among high-risk groups.

## Figures and Tables

**Table 1:** Demographics of serum specimens.

Characteristic	N	Female, N = 10,308 ^1^	Male, N = 10,057 ^1^	Overall ^1^
**Age Group**	20,365			
1 to 11		1443 (14%)	1483 (15%)	2926 (14%)
12 to 19		2040 (20%)	1843 (18%)	3883 (19%)
20 to 39		2409 (23%)	2180 (22%)	4589 (23%)
40 to 59		2248 (22%)	2298 (23%)	4546 (22%)
60+		2168 (21%)	2253 (22%)	4421 (22%)
**Age**	20,365			
Median (IQR)		33 (17, 56)	36 (17, 58)	34 (17, 57)
Range		1, 102	1, 101	1, 102
**Vaccinated before Specimen Collection**	20,365	5194 (50%)	5228 (52%)	10,422 (51%)
**First Dose**	16,558			
AstraZeneca		420 (5.0%)	598 (7.3%)	1018 (6.1%)
Moderna		1715 (20%)	1911 (23%)	3626 (22%)
Pfizer		5599 (67%)	4973 (61%)	10,572 (64%)
Other		657 (7.8%)	685 (8.4%)	1342 (8.1%)
**Second Dose**	15,840			
Moderna		2392 (30%)	2737 (35%)	5129 (32%)
Pfizer		5110 (63%)	4480 (58%)	9590 (61%)
Other		551 (6.8%)	570 (7.3%)	1121 (7.1%)
**Third Dose**	8701			
Moderna		761 (18%)	884 (20%)	1645 (19%)
Pfizer		2728 (63%)	2625 (60%)	5353 (62%)
Moderna Bivalent		65 (1.5%)	72 (1.6%)	137 (1.6%)
Pfizer Bivalent		115 (2.7%)	139 (3.2%)	254 (2.9%)
Other		631 (15%)	681 (15%)	1312 (15%)
**Fourth Dose**	4236			
Moderna		103 (5.0%)	149 (6.9%)	252 (5.9%)
Pfizer		842 (41%)	909 (42%)	1751 (41%)
Moderna Bivalent		445 (21%)	502 (23%)	947 (22%)
Pfizer Bivalent		529 (26%)	418 (19%)	947 (22%)
Other		154 (7.4%)	185 (8.6%)	339 (8.0%)
**Fifth Dose**	1618			
Moderna		3 (0.4%)	5 (0.6%)	8 (0.5%)
Pfizer		8 (1.0%)	14 (1.6%)	22 (1.4%)
Moderna Bivalent		257 (34%)	310 (36%)	567 (35%)
Pfizer Bivalent		489 (64%)	501 (59%)	990 (61%)
Other		8 (1.0%)	23 (2.7%)	31 (1.9%)
**Sixth Dose**	129			
Moderna		0 (0%)	1 (1.4%)	1 (0.8%)
Pfizer		0 (0%)	0 (0%)	0 (0%)
Moderna Bivalent		2 (3.3%)	3 (4.3%)	5 (3.9%)
Pfizer Bivalent		52 (87%)	55 (80%)	107 (83%)
Other		6 (10%)	10 (14%)	16 (12%)
**Total Doses Received**	20,365			
0		1917 (19%)	1890 (19%)	3807 (19%)
1		338 (3.3%)	380 (3.8%)	718 (3.5%)
2		3753 (36%)	3386 (34%)	7139 (35%)
3		2227 (22%)	2238 (22%)	4465 (22%)
4		1308 (13%)	1310 (13%)	2618 (13%)
5		705 (6.8%)	784 (7.8%)	1489 (7.3%)
6		60 (0.6%)	69 (0.7%)	129 (0.6%)
**RHA**	20,365			
IEHR		976 (9.5%)	956 (9.5%)	1932 (9.5%)
NHR		847 (8.2%)	802 (8.0%)	1649 (8.1%)
PMHR		1230 (12%)	1177 (12%)	2407 (12%)
SHSS		1447 (14%)	1376 (14%)	2823 (14%)
WRHA		5808 (56%)	5746 (57%)	11,554 (57%)
**Anti-Spike IgG**	14,089			
Negative		2382 (34%)	2385 (34%)	4767 (34%)
Positive		4667 (66%)	4655 (66%)	9322 (66%)
**Anti-Nucleocapsid IgG**	20,365			
Negative		6567 (64%)	6246 (62%)	12,813 (63%)
Positive		3741 (36%)	3811 (38%)	7552 (37%)
**Positive for Anti-Spike and Anti-Nucleocapsid IgG**	20,365	2176 (21%)	2167 (22%)	4343 (21%)
**Positive for Anti-Spike IgG Only**	20,365	2491 (24%)	2488 (25%)	4979 (24%)
**Weeks between First Dose and Specimen Collection**	10,561			
Median (IQR)		38 (20, 56)	37 (20, 54)	38 (20, 55)
Range		0, 117	0, 116	0, 117
**Weeks between First and Second Dose**	15,840			
Median (IQR)		7.0 (5.0, 10.0)	7.0 (5.0, 10.0)	7.0 (5.0, 10.0)
Range		3.0, 65.0	3.0, 78.0	3.0, 78.0
**Weeks between Second Dose and Specimen Collection**	9147			
Median (IQR)		35 (19, 51)	34 (19, 49)	34 (19, 50)
Range		0, 114	0, 113	0, 114
**Weeks between Second and Third Dose**	8701			
Median (IQR)		28 (27, 33)	28 (26, 32)	28 (26, 33)
Range		4, 109	4, 98	4, 109
**Weeks between Third Dose and Specimen Collection**	3432			
Median (IQR)		19 (9, 36)	18 (9, 36)	19 (9, 36)
Range		0, 78	0, 78	0, 78

^1^ n (%).

## Data Availability

The data analyzed in this study is subject to the following licenses/restrictions: data are not available as they cannot be shared outside of the Government of Manitoba. Requests to access these datasets should be directed to Derek R. Stein (Derek.Stein@gov.mb.ca) or Jason Kindrachuk (Jason.Kindrachuk@umanitoba.ca).

## Supplementary Materials

The following supporting information can be downloaded at: https://www.mdpi.com/article/10.3390/vaccines12101095/s1, Table S1: Summary of COVID-19 vaccine eligibility changes in Manitoba; Figure S1: Sex differences in SARS-CoV-2 anti-spike IgG after one or two doses of a COVID-19 vaccine; Figure S2: Differences in SARS-CoV-2 anti-spike IgG by regional health authority after one or two doses of a COVID-19 vaccine.
