# Supplementary material for: A Population-Based Study of SARS-CoV-2 IgG Antibody Responses to Vaccination in Manitoba
Source: Vaccines (Basel). 2024 Sep 26;12(10):1095. doi: 10.3390/vaccines12101095 (PMC11511381; doi:10.3390/vaccines12101095)
Supplement: Supplementary file 1 [file vaccines-12-01095-s001.zip › vaccines-3012396-supplementary.pdf]

Table S1. Summary of COVID-19 vaccine eligibility changes in Manitoba.

Date	Eligibility Changes	Organization	Dose
20/12/09	Pfizer BNT162b2 approved for age 16+	Health Canada	NA
20/12/23	Moderna mRNA-1273 authorized for people 18+	Health Canada	NA
21/02/24	Age 95+; First Nations age 75+	Government of Manitoba	1
21/02/26	Authorized AstraZeneca vaccine for people 18+	Health Canada	NA
21/03/08	Age 80+; First Nation people age 60+	Government of Manitoba	1
21/03/15	Age 50-64; First Nations age 30-64 eligible for AstraZeneca vaccine	Government of Manitoba	1
21/03/17	Age 73+; First Nations age 53+	Government of Manitoba	1
21/03/22	Age 65+; First Nations age 45+	Government of Manitoba	1
21/03/29	Age 64+; First Nations age 44+	Government of Manitoba	1
21/04/07	Age 62+; First Nations age 42+	Government of Manitoba	1
21/04/14	Age 59+; First Nations age 39+	Government of Manitoba	1
21/04/21	50+ and First Nations 30+; front-line police and firefighters	Government of Manitoba	1
21/05/03	All Indigenous people 18+	Government of Manitoba	1
21/05/05	Age 45+	Government of Manitoba	1
21/05/05	Authorized Pfizer BNT162b2 vaccine in age 12-15	Health Canada	NA
21/05/07	Age 40+	Government of Manitoba	1
21/05/10	Age 30+	Government of Manitoba	1
21/05/12	Age 18+	Government of Manitoba	1
21/05/14	Ages 12-17 eligible for Pfizer BNT162b2	Government of Manitoba	1
21/05/21	Priority health conditions	Government of Manitoba	2
21/05/24	Indigenous people	Government of Manitoba	2
21/05/25	All Indigenous people; individuals with specific health conditions	Government of Manitoba	2

Date	Eligibility Changes	Organization	Dose
21/06/25	All 18+	Government of Manitoba	2
21/08/16	All 12+	Government of Manitoba	2
21/09/20	All residents and staff of First Nation PCHs	Government of Manitoba	3
21/10/06	Viral vector vaccine recipients; First Nation health care workers and residents	Government of Manitoba	3
21/10/18	All First Nations living on reserves	Government of Manitoba	3
21/10/27	All PCH residents	Government of Manitoba	3
21/11/03	Age 70+; First Nation, Inuit and Métis age 18+	Government of Manitoba	3
21/11/15	All 18+	Government of Manitoba	3
21/11/19	Age 5-11 eligible for Pfizer vaccine	Government of Manitoba	1
21/12/24	Age 50+	Government of Manitoba	3
22/04/06	Age 70+; PCH residents; First Nations, Inuit and Métis age 50+	Government of Manitoba	4
22/04/13	Age 12 to 17 with medical conditions who belong to racialized/marginalized communities or who live in shelters/group homes	Government of Manitoba	3
22/05/20	All 18+	Government of Manitoba	4

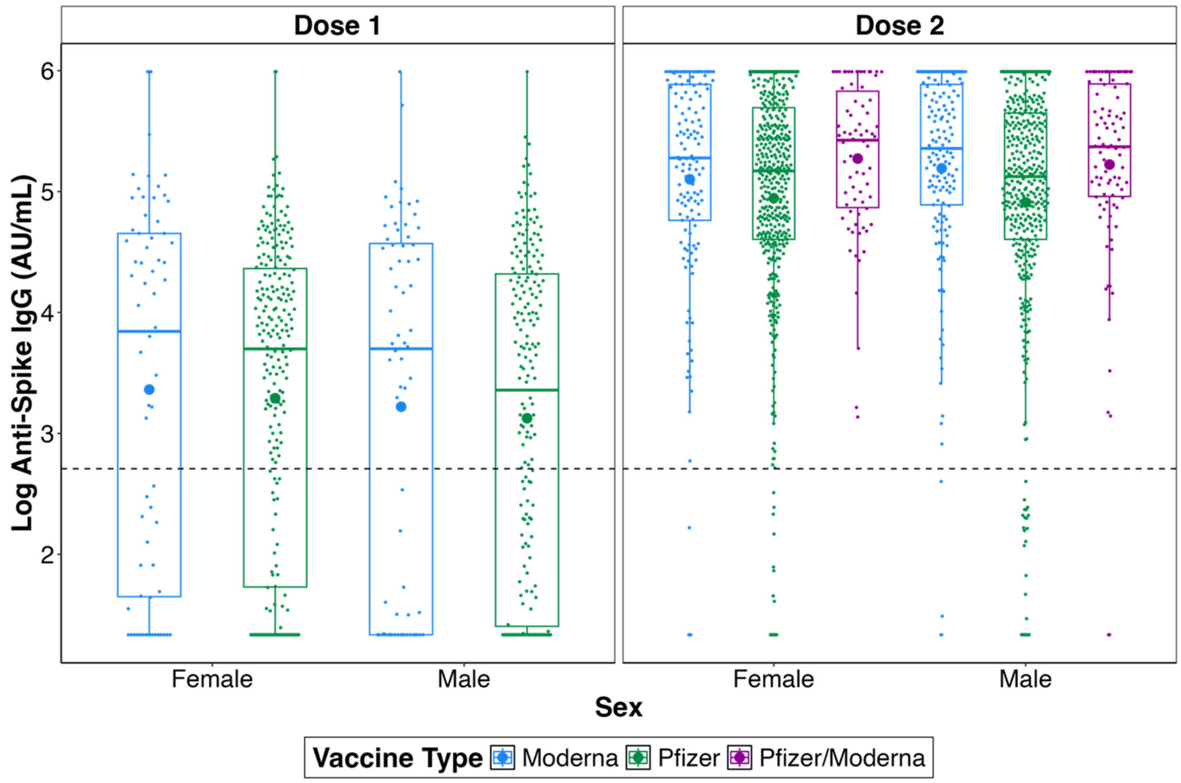

Figure S1. Sex differences in SARS-CoV-2 anti-spike IgG after one or two doses of a COVID-19 vaccine. Only samples negative for anti-nucleocapsid IgG were included. Specimens were vaccinated with either one dose of Pfizer BNT162b2 (green) or Moderna mRNA-1273 (red), or with two doses of Pfizer BNT162b2 (green) or Moderna mRNA-1273 (red), or one dose of each (Pfizer/Moderna) (blue). Dunn's test for pairwise comparisons corrected for multiple hypothesis testing using the Benjamini-Hochberg method was used to compare groups. No significant differences were found. Larger dots are the mean. Smaller dots represent individual samples.

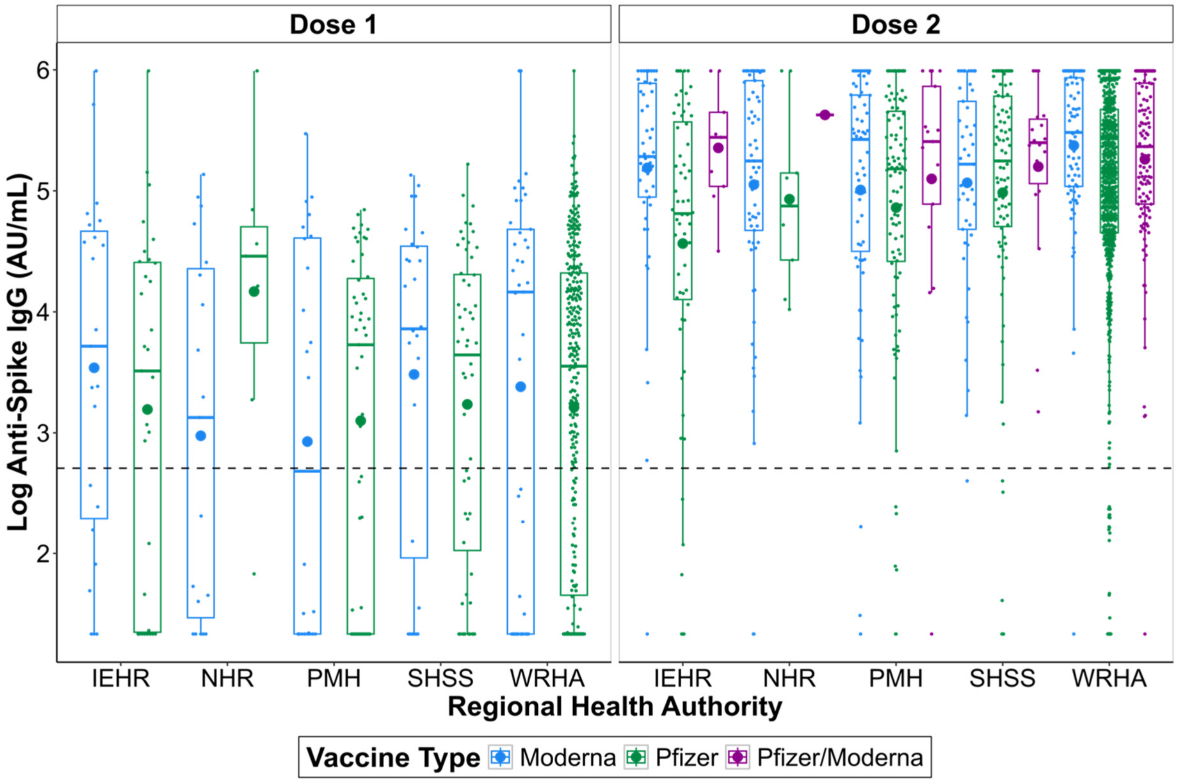

Figure S2. Differences in SARS-CoV-2 anti-spike IgG by regional health authority after one or two doses of a COVID-19 vaccine. Only samples negative for anti-nucleocapsid IgG were included. Specimens were vaccinated with either one dose of Pfizer BNT162b2 (green) or Moderna mRNA-1273 (red), or with two doses of Pfizer BNT162b2 (green) or Moderna mRNA-1273 (red), or one dose of each (Pfizer/Moderna) (blue). Dunn's test for pairwise comparisons corrected for multiple hypothesis testing using the Benjamini-Hochberg method was used to compare groups. No significant differences were found. Larger dots are the mean. Smaller dots represent individual samples. IEHR, Interlake-Eastern Health Region; NHR, Northern Health Region; PMH, Prairie Mountain Health; SHSS, Southern Health Santé Sud; WRHA, Winnipeg Regional Health Authority.
